# Self-Reported Hearing-Aid Use Patterns in an Adult Danish Population

**DOI:** 10.3390/audiolres13020021

**Published:** 2023-03-27

**Authors:** Sreeram K. Narayanan, Sabina S. Houmøller, Anne Wolff, Katja Lund, Sören Möller, Dan D. Hougaard, Michael Gaihede, Jesper H. Schmidt, Dorte Hammershøi

**Affiliations:** 1 Section for AI and Sound, Department of Electronic Systems, Aalborg University, 9220 Aalborg, Denmark; 2 Research Unit for ORL—Head & Neck Surgery and Audiology, Odense University Hospital, 5230 Odense, Denmark; 3 OPEN (Open Patient Data Explorative Network), Odense University Hospital, 5000 Odense, Denmark; 4 Department of Clinical Research, University of Southern Denmark, 5230 Odense, Denmark; 5 Department of Otolaryngology, Head & Neck Surgery and Audiology, Aalborg University Hospital, 9000 Aalborg, Denmark; 6 Department of Clinical Medicine, Aalborg University, 9000 Aalborg, Denmark

**Keywords:** hearing aid usage pattern, self-reported outcome, HA rehabilitation, hearing aid

## Abstract

The retrospective reporting of users’ hearing aid (HA) usage can provide insight into individualized HA usage patterns. Understanding these HA usage patterns can help to provide a tailored solution to meet the usage needs of HA users. This study aims to understand the HA usage pattern in daily-life situations from self-reported data and to examine its relationship to self-reported outcomes. A total of 1537 participants who responded to questions related to situations where they always took off or put on the HAs were included in the study. A latent class analysis was performed to stratify the HA users according to their HA usage pattern. The results showed distinct usage patterns in the latent classes derived for both scenarios. The demographics, socio-economic indicators, hearing loss, and user-related factors were found to impact HA usage. The results showed that the HA users who reported using the HAs all the time (regular users) had better self-reported HA outcomes than situational users, situational non-users, and non-users. The study explained the underlying distinct HA usage pattern from self-reported questionnaires using latent class analysis. The results emphasized the importance of regular use of HAs for a better self-reported HA outcome.

## 1. Introduction

Hearing aid (HA) use is highly individual and, among other things, is determined by listening intent and exposed acoustic environment [1]. For example, some may use their HAs only for attending to certain tasks in a specific acoustic environment, such as watching television at home, and not wear them during sleep. Previous studies suggest that HA use may also be driven by factors such as the degree of hearing loss (HL), cognitive abilities, higher motivation towards HA use, prior experience [2,3], and external expectations (relatives, employers, etc.). This behavior of the HA users results in individualized usage patterns. HA users who intend to communicate in complex listening situations such as large gatherings or noisy environments may achieve less benefit than HA users with less complex communication needs [4]. So, understanding HA users’ listening lifestyles and patterns of use can provide insights that can help clinicians provide personalized care and counseling, which may improve the overall quality of hearing care [5,6].

The daily use of HAs varies across HA users [1,7]. Even though a higher use of HAs seemed beneficial [4], it may not be true in all cases [8,9]. Laplante-Levesque et al. [10] examined the usage pattern of HA users and found two types of HA usage behavior. One set of HA users wore their HAs all day, from waking up in the morning to going back to sleep in the evening; the other set of HA users used their HAs strictly on a need basis. A recent study also analyzed HA usage patterns and found four clusters of HA users according to their usage patterns [6]. The HA users in Cluster 1 used their HAs in the morning; the Cluster 2 HA users used them in the evening; users in Cluster 3 had intermittent usage during the whole day; and Cluster 4 used their HAs consistently for the whole duration of the day. Christensen et al. [6] also found a significant difference in usage according to the day of the week.

Humes et al. [11] investigated the acoustic environments in which older HA users wore their HAs from the data obtained from the HA data logging. The results showed that 60% of the HA users wore their HAs in quiet and in a moderate level of speech-only situations and found that the HA users least wore their HAs in high-level (≥75 dB SPL) speech-in-noise situations. These results were consistent in the short-term (6 weeks) and long-term (1–2 years) HA use. Andersson et al. [12] also examined HA usage using Ecological Momentary Assessment (EMA) and found that 49% of the HA users used their HAs in quiet conditions, 39% in speech-only conditions, 8% used them in speech-in-noise situations, and only 4% used it in noisy conditions. The results were consistent when compared with the HA data-loggings.

Most previous studies have focused on understanding the usage pattern in terms of HA usage time and the exposed acoustic environments while using HAs. The acoustic data extracted from HA data loggings do not capture the context of HA usage [12]. The contextual information provides insight into the intention of HA use, which can be one of the determinants of user preferences. In this study, we try to understand usage patterns through retrospective reports of the HA users related to their HA usage. We collected retrospective reports of use (situations where HAs were always taken off or worn) at a point in time (at least one year after fitting) where regular HA usage could be assumed. The present study tries to understand any common underlying patterns of HA usage derived from the situations of use and non-use amongst the participants. It also combines self-reported HA usage and outcome with socio-demographic, auditory, and HA-related factors to identify any underlying sub-populations that may need clinical attention. It is hypothesized that the HA users may exhibit distinct HA usage patterns that depend on demographic, audiologic, and HA-related characteristics and that the usage patterns affect efficacy and benefit, which in turn will be reflected in the self-reported HA outcomes.

## 2. Materials and Methods

In Denmark, patients have the choice to receive HAs funded by the Danish government. The patients are offered HAs through public and private clinics, including the necessary repair and maintenance. Data used in the study were collected at the Departments of Audiology at Odense University Hospital, Region of Southern Denmark, and Aalborg University Hospital, Region of Northern Denmark. A total of 1961 patients agreed to participate in the data collection. The inclusion criteria were that they should be adults with serviceable hearing loss who could read and understand Danish. The patients were excluded if they were a candidate for a cochlear implant or bone-anchored hearing aids, candidates for other surgical treatments of hearing loss, had malfunctioning auricles or inner ear, or had tinnitus without concurrent clinically diagnosed hearing loss [13]. The details about the data collection protocol can be found in Wolff 2019 [13] and Houmøller 2021 [14].

The data were collected in three stages: baseline, two months after HA fitting (two-month follow-up with in-clinic consultation), and more than one year after fitting (one-year follow-up questionnaires without consultation). The participants were sent a digital notice (through e-boks; https://www.e-boks.com/danmark/en; accessed on 30 January 2023) with a link to questionnaires, which, among others, included two non-standardized questions relating to situational use. Two independent questions were asked: (1) “Are there any situations where you always take off your hearing aids?” and (2) “Are there any situations where you always put your hearing aids on?”. The original Danish wording would imply taking them off or putting them on physically (not just turning them on or off). In both cases, the first response option was, “No, I pretty much always wear them.” If not choosing this option, in either case, the same ten alternative response options were shown in a check-all-that-apply format, enabling HA users to select as many situations for use or non-use as they considered relevant. The ten alternative response options were nine daily-life situations, namely: while watching television (TV), while shopping (shopping), while walking (walking), while cycling (cycling), in church (church), while using the telephone (telephone), at home (home), while driving (driving), at work (work), and finally the other (other) option, giving free-text response opportunity. The HA users who selected the first option can be considered regular users of the HAs, and those who chose to respond with different situations that they put on or took off their HAs can be considered situational users of the HAs.

In the one-year follow-up, we collected data on the socio-economic (marital status, number of members in the household, education, and income) conditions of the participants, as well as self-reported outcomes through, among others, the abbreviated version of the Speech, Spatial, and Quality of Hearing (SSQ12) [15] and the International Outcome Inventory for Hearing Aids (IOI-HA) [16,17]. The SSQ12 comprised 12 questions representing three domains. The speech domain has five questions, the spatial domain has three, and quality has four questions in SSQ12. The IOI-HA has seven items (use of HA, perceived benefits, residual activity limitation, satisfaction, residual participation restriction, impact on others, and quality of life) representing recent experiences regarding the effectiveness of HA rehabilitation. Factor analysis of IOI-HA showed two significant factors; Factor 1 (introspection: use of HA, perceived benefits, satisfaction, and quality of life) and Factor 2 (interaction: residual activity limitation, residual participation restriction, and impact on others) [16].

### 2.1. Grouping of the “Other” Situations

The description of the “other” situations where the participants preferred to take off or put on their HAs was categorized and labeled by three researchers (independently), followed by two consensus-forming joint brainstorming sessions, see Table 1. Eleven categories were identified and labeled from the free-text description of situations where the HA users reported always taking their HAs off. Four categories were identified and labeled from the free-text description of situations where HA users always reported using their HAs. The categories pertaining to cultural and outdoor activity were common for both scenarios. These labeled categories of situations derived from free-text descriptions of the situations, along with a short description of each category, are detailed in Table 1.

It was found that some of the HA users in the description said that they no longer used their HAs. These users were treated separately as “non-users.” There were 69 HA users who provided invalid responses in the descriptions. Out of 69, 58 HA users clicked only on the “other” tab and did not provide any detailed response. Five HA users responded that they always used their HAs and had checked on the box for other options but did not provide detailed comments. The remaining six were HA users who either had put tick marks on all the situations for both questions, which meant they were just clicking on each response option to finish the questionnaire or provided precisely the same responses to both questions. These data were excluded from further analysis.

### 2.2. Sample

Out of 1961 who participated in the study, 1537 participants responded to both questions. For the questions related to situations where participants took off their HAs, 655 (43%) participants responded that they pretty much always wore their HAs (regular users), and 882 (57%) responded with situations where they always chose to take off their HAs (situational non-users). Out of 882, there were 27 (3%) invalid responses, and 82 (9%) using the free-text description reported that they were not using their HA anymore. The non-users of HA were treated as a separate group. A total of 874 participants (410 regular users; 426 situational non-users; 38 non-users) out of 1537 who had valid socio-demographic, audiological, and self-reported outcome data were included in the further analysis with regards to the situations where participants always chose to take off their HAs (see overview in Figure S1 of the Supplementary Materials).

Regarding the question about situations where the participants always put on their HAs, 914 (59%) out of 1537 reported regular use, of which only 573 had valid socio-demographic, audiological, and self-reported outcome data. A total of 623 participants reported situational use of the HA; out of that, 66 were non-users, and 49 had invalid responses. There were 271 situational users and 24 non-users with valid socio-demographic, audiological, and self-reported outcome data. A total of 868 participants were included in the further analysis of situations where participants always chose to put on their HAs (see overview in Figure S2 of the Supplementary Materials). Out of the HA users with valid data, 174 reported situations where they would always take off their HAs and also reported they would always put on the HAs in response to the question related to situations where they always put on their HAs.

Out of 38 and 24 non-users identified above from both questions, 18 participants responded similarly to both questions, whereas other users responded differently. Twenty participants responded that they were either not using or sparingly using the HAs to the question related to the situation where they always took off their HAs and responded with those situations where they sparingly used the HAs in response to the question related to situations where they always put on their HAs. In addition, six participants responded with situations where they always took off their HAs and stated that they used hearing aids sparingly for the question related to situations where they always put on their HAs.

### 2.3. Statistical Analysis

The data were managed and analyzed using R software version 4.2.1 and RStudio version 2022.07.1 (RStudio, Inc., Boston, MA, USA). A Latent Class Analysis (LCA) [18] was performed, assuming that latent classes could explain patterns of observed responses to different situations. LCA is used to identify sub-groups within a population that may have similar characteristics [19]. The analysis was performed using the poLCA package [20] available in R. Like many other clustering methods, LCA requires the intended number of latent classes to be specified a priori. To select and validate the optimal number of latent classes and to maximize model fit, models of up to six latent classes were compared based on standard test statistics. The test statistics included were the Bayesian Information Criterion (BIC), the Akaike Information Criteria (AIC), the Consistent Akaike Information Criterion (CAIC), and the bootstrap likelihood ratio test (BLRT). We also examined the model fit by comparing the variable-specific entropy contribution. The latent class models computed with up to six latent classes for comparing model parameters to determine the optimal number of latent classes only included the responses to situations and not the covariates. After determining the optimal number of latent classes, the model was computed again, including the covariates. The covariates included in the analysis were age, gender, average four-frequency (500 Hz, 1 kHz, 2 kHz, and 4 kHz) pure-tone air-conduction hearing threshold of the better hearing ear (PTA4), word recognition score of the better hearing ear (WRS), prior experience using HA (first-time or experienced), PTA-based asymmetric HL defined as PTA4 difference of higher than 10 dB between best and worst hearing ear, the presence of tinnitus indicated by the Tinnitus Handicap Inventory (THI) score, average score to the two motivation-related questions from the tool developed by the Ida Institute (www.idainstitute.com; accessed on 20 January 2023), current work status, number of household members, and income. The covariates did not include any aided performance measures, as the same was not recorded as part of the data collection. The data collection was observational in nature, reviewing the current clinical practices in Denmark. As aided measures are not part of the current clinical practice, the same was not recorded.

The predicted latent class membership was used as the indicator of situational use/non-use of the HA. We used the Kruskal–Wallis and chi-squared tests to understand the differences between the regular users, situational users/non-users, and non-users concerning each covariate. The Kruskal–Wallis test was used for testing continuous variables and chi-squared for categorical variables. Finally, a stepwise multiple linear regression [21] using forward selection and backward elimination was used to understand the effect of class membership on self-reported outcomes. We adjusted the model for all the covariates included in the study. The IOI-HA scores were weighted using the weights developed by Leijon et al. [22] before calculating the two factor scores. The SSQ12 total scores were calculated by averaging the responses to each question in the questionnaire. Separate models were created for each IOI-HA factor and SSQ12 total score.

## 3. Results

### 3.1. Situational Non-Use and Situational Use of HAs

**Figure 1:**
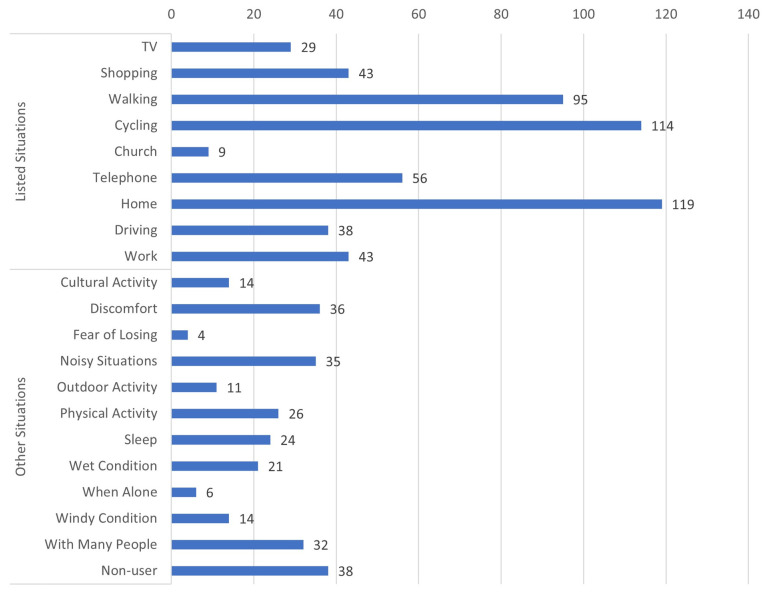
Situations where HA users always took off their HAs. The numbers beside the bar are the number of participants responding to the given specific situation where they always took off their HAs. The categories of situations listed from “TV” to “Work” were the listed situations, and the rest from “Cultural Activity” to “With Many People” are categories derived from the theme analysis of the free-text responses delivered by the HA users using the “other” option.

Figure 1 shows 807 responses from 464 participants across categories of situational non-use (when they would always take them off). This included 38 participants who said they no longer/sparingly used their HAs. This group was treated separately in further analysis. It can be seen that 119 reported always taking their HAs off when at home, 95 when walking, and 114 when cycling. In addition, many reported always taking their HAs off while talking on the telephone (streaming options not available in the HA technology used), while driving, at work, watching TV, shopping, or even when with many people.

**Figure 2:**
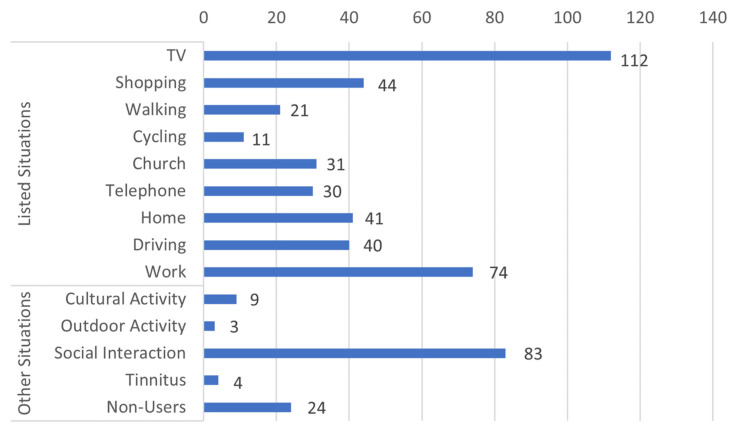
Situations where HA users always put on their HAs. The numbers beside the bar represent the number of participants responding to a specific situation where they always put on their HAs. The situations listed in the questions are in the “listed situation” group, and the “other situation” group are situations that are derived from the theme analysis of the free-text responses delivered by the HA users using the “other” option.

Figure 2 shows 527 responses from 295 participants across categories of situational use (when they would always put their HAs on). There were 24 HA users who responded to the same question saying they no longer use their HAs. These HA users were treated separately in a non-user group in further analysis. From Figure 2, it can be seen that the participants reported always putting their HAs on predominantly in three situations; (1) while watching television (112 responses), (2) when at work (74 responses), and (3) during social interaction (83 responses).

### 3.2. Fitting the Latent Class Models

The LCA models with 2 to 6 classes were fitted and examined to determine the optimal number of latent classes across situational non-use/use categories. The non-user group was not included in the LCA as these responses were not a specific situation, and in an LCA, they would emerge as a separate class anyway. The tables showing the model fit statistics for both scenarios can be found in Tables S1 and S2 of the Supplementary Materials. The statistical model fitting indicators were inconclusive for both scenarios. In both cases, the BIC and CAIC favored the 2-class model; however, the AIC favored models with higher classes. The BLRT provided evidence for a 6-class model for situations where HA users reported always taking their HAs off and for a 5-class model for situations where HA users reported always putting their HAs on, as indicated by a *p*-value < 0.05 for these models. Nevertheless, considering the sample size of 271 and 426, we thought that dividing them into 5 or 6 classes would result in classes with very few members. So, we resorted to a 3-class model in both cases, with the statistical indicator (AIC, CAIC, and BIC) not significantly different from the 2-class model. The entropy values that indicate good separation of the classes were 0.83 and 0.95, respectively, for situations where participants took off and put on their HAs, which was above the ideal mark of 0.8 [23]. The optimal model was recomputed, including the covariates.

### 3.3. Fitted 3-Class Latent Class Model

**Figure 3:**
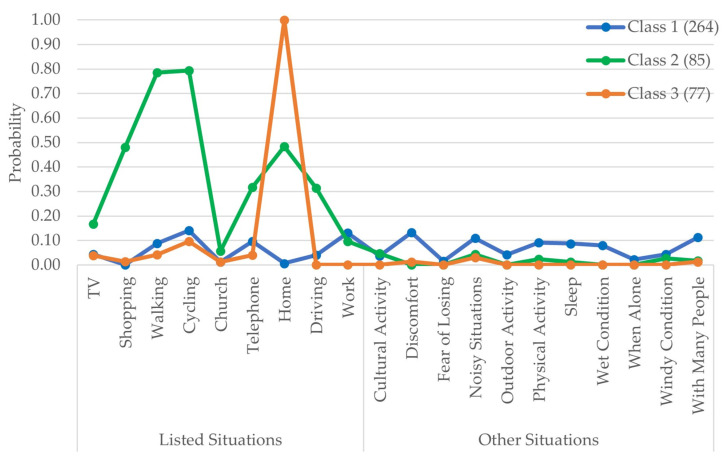
The item response probabilities for situations where HA users always took off their HAs with respect to each latent class. The legend also shows the class proportions. The situations listed in the questions are in the “listed situation” group, and the “other situation” group are situations that are derived from the theme analysis of the free-hand text responses delivered by the HA users using the “other” option.

Figure 3 shows the class proportions and item response probabilities of situations where the participants reported always taking their HAs off. Class 1 comprised approximately 62% (264 participants) of the respondents with the least probability of taking off the HAs in situations relating to shopping, church, home, cultural activity, fear of losing, outdoor activity, and when alone. The probabilities of taking off the HAs ranged from 5 to 15% in situations related to TV, walking, cycling, telephone, work, discomfort, noisy situations, physical activity, sleep, wet conditions, windy conditions, and situations with many people. Class 2, comprising 20% (85) of the participants, had a higher probability of reporting taking off their HAs in situations such as shopping, walking, cycling, using the telephone, at home, and driving. Class 3 had 18% (77) of participants who reported always taking off their HAs at home.

**Figure 4:**
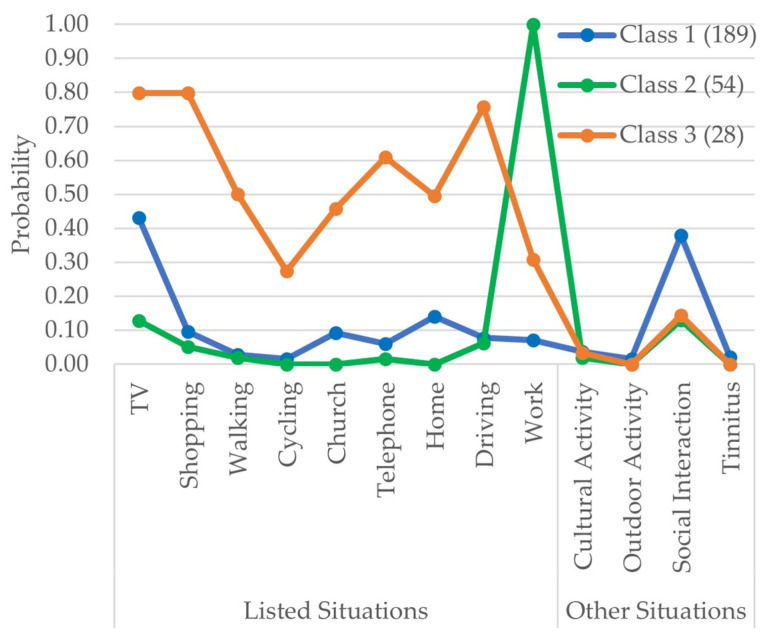
The item response probabilities for situations where HA users always preferred to put on their HAs with respect to each latent class. The legend also shows the number of participants in each class. The situations listed in the questions are in the “listed situation” group, and the “other situation” group are situations that are derived from the theme analysis of the free-hand text responses delivered by the HA users using the “other” option.

Figure 4 shows the class proportion and probabilities of situations where participants reported always putting their HAs on. The participants with membership in Class 1 were the majority, with 70% of the respondents (*n* = 189) and had a higher probability of reporting putting the HAs on for watching TV and during social interactions. Class 2 had 54 participants and a higher probability of reporting putting the HAs on for work and watching TV. Class 3 was the smallest in proportion, with approximately 10% (*n* = 28) of the participants. The HA users in this class had more than a 60% probability of using their HAs while watching TV, shopping, using the telephone, and driving.

### 3.4. Covariates

The distribution of covariates across participants stratified according to the HA usage (regular, latent classes of situational non-use/use, and non-use) are shown in Tables S3 and S4 of the Supplementary Materials. In the case of situations where HA users reported always taking off their HAs (Table S3 of the Supplementary Material), the distribution of males and females across the classes of participants was significantly different. There were significantly more females in the class of users with a higher probability of taking the HA off when at home (Class 3) than in other classes. The regular users and non-users had similar gender proportions, which was also significantly different from the class of situational non-users who had the least probability of taking off their HAs in multiple situations (Class 1) and the class of participants who had a higher probability of taking off their HAs in situations such as shopping, walking, cycling, on the telephone, at home, and while driving (Class 2), with a higher proportion of male participants. The proportion of first-time users in Class 2 of situational non-users was also significantly higher than in other classes. Class 2 of situational non-users and non-users had significantly better mean better hearing ear PTA4 than other classes. Class 2 also had a higher proportion of interlateral asymmetry, and Class 3 (higher probability of taking the HA off at home) had a higher proportion of participants with symmetric hearing than other classes. The baseline motivation toward HA rehabilitation indicated by scores for the two questions shows a significantly lower mean score for the participant in the class with a higher probability of taking off the HAs in multiple situations (Class 2) and non-users. There was no significant difference between the classes concerning the current work status of the participants and the number of household members living with the participants, including themselves. Compared to other classes, Class 2 had a higher proportion of HA users with a yearly income between DKK 300,000 and 600,000 (EUR 40,000 and 80,000). The probability of participants with an income from DKK 600,000 to 900,000 (EUR 80,000 to 120,000) to be a member of Class 2 (higher probability of taking off the HAs in multiple situations) was lower than other classes. The Class 3 (higher probability of taking off the HA at home) participants were more likely to have an income higher than DKK 900,000 (EUR 120,000) than other classes.

Table S4 the Supplementary Materials shows the effect of covariates on the class membership of participants who responded to questions related to the situational use of the HA. Participants with membership in the class with a higher probability of putting on the HAs at work (Class 2) were significantly younger than the other classes and had (as would be expected) a significantly better mean PTA4 for the better hearing ear than other classes. There was a significantly higher proportion of experienced HA users in Class 3 (higher probability of putting on the HAs in most of the listed situations) of the situational users and among regular users than in other classes. The motivation scores about the readiness toward HA rehabilitation of participants with membership in the class of situational users with a higher probability of putting on the HAs for watching TV and for social interactions (Class 1) were lower than participants in other classes. The non-users and Class 1 situational users also reported a lower motivation score about self-efficacy. The participants in Class 2 (higher probability of putting on the HAs at work) were more likely to be working than other classes. Class 1 (higher probability of putting on the HAs for watching TV and for social interactions) of situational users had a significant proportion of participants who lived alone or in a household of three or more members. The participants with membership in Class 2 were more likely to have a yearly income between DKK 300,000 and 900,000 (EUR 40,000 and 120,000), and regular users were more likely to have an income between DKK 100,000 and 600,000 (EUR 13,000 and 80,000). Class 2 also had a significantly higher proportion of people with an income of more than DKK 900,000 (EUR 120,000).

### 3.5. Self-Reported Outcome

The result of stepwise multiple linear regression showing the effect of HA usage patterns and other covariates on self-reported outcomes (IOI-HA Factor 1, IOI-HA Factor 2, and SSQ12) is shown in Table 2 and Table 3. The effects of the covariates on the self-reported outcome were very similar, barring minimal changes in the effect sizes for both scenarios (putting on or taking off of HAs), even though the number of participants responding to the situations where they always took off or put on their HAs was different. It is also fair to observe that both sets of participants come from the same pool of participants, and this result can be expected. The result also shows a significant effect of non-use, situational use, and situational non-use of HAs compared to regular use on the self-reported outcome.

#### 3.5.1. Effect of Situational Use on Self-Reported Outcomes

From Table 2, for the question regarding situations where they always took off their HAs, the situational non-users (Class 1, Class 2, and Class 3) and the participants who reported that they were not using their HAs anymore had a significantly lower IOI-HA Factor 1 score compared to participants who reported that they were regular users. Similar results were seen for the latent classes of situational use of HAs and non-users derived from the responses to questions about the situations where they always put on the HAs. Compared to regular users, participants with membership in Class 1 and Class 2 of situational non-use and non-users reported a significantly lower IOI-HA Factor 2 score. At the same time, participants with membership in Class 1 and Class 3 of situational use and non-users also reported a significantly lower Factor 2 score than regular users. The participants who preferred to take off their HAs while walking and cycling (Class 2) and those who always took off their HAs at home (Class 3) reported significantly lower total SSQ12 scores than regular users. The participants who preferred to put on their HAs while watching TV or during social interaction (Class 1), the participants who were more likely always to put on their HAs while watching TV, shopping, on the telephone, and driving (Class 3), and non-users also reported a lower SSQ12 total score than regular users.

#### 3.5.2. Effect of Other Variables on Self-Reported Outcomes

Males reported a better SSQ12 total score than females after more than one year of HA usage. First-time HA users reported better IOI-HA Factor 2 and SSQ12 total scores compared to HA users with prior experience using a HA. Participants with higher deficits (poorer PTA4 in the better-hearing ear) reported higher Factor 1 scores but significantly lower Factor 2 and SSQ12 total scores. Participants with PTA-based asymmetric HL (PTA4 difference higher than 10 dB between best and worst hearing ears) reported lower self-reported outcomes (Factor 1, Factor 2, and SSQ12 total scores) than participants with symmetric PTA4 scores. Participants who reported having tinnitus also reported lower Factor 2 and SSQ12 total scores. The motivation in terms of readiness toward HA rehabilitation significantly affected Factor 2 scores. The results showed that participants with higher self-efficacy (Motivation Q2) reported higher IOI-HA and SSQ12 scores. Not working participants reported a lower Factor 2 score than the working participants. Participants with an income of DKK 900,000 (EUR 120,000) or more reported a higher SSQ12 total score than participants between DKK 100,000 to 300,000 (EUR 13,000 to 40,000). In contrast, the participants with an income under DKK 100,000 (EUR 13,000) reported a lower SSQ12 total score.

## 4. Discussion

In the present study, 655 HA users out of 1537 respondents consistently reported always using their HAs, corresponding to 41%. This is significantly lower than the 57% and 64% reported by Laplante-Lévesque et al. [10] and Pasta et al. [5], respectively. The raw data (bar plots in Figure 1 and Figure 2) showed distinct use patterns, reporting more situations where they would always take the HAs off than the opposite (put them on). This could reflect that the majority would default to wearing them but occasionally take them off. It could also be because of the human tendency to recollect unfulfilling situations more than fulfilling ones, which is well established by Kensinger [24].

The LCA using the responses of the HA users to situations where they always took off their HAs shows that Class 1 had 264 HA users with significantly less item response probability of taking off the HAs in most of the listed situations. Class 2 were HA users who were selectively using their HAs for specific situations, as there were many listed situations where the item response probabilities were higher, indicating the possibility of HA users not using the HA in those situations. Class 3 was particular as it contained HA users who did not use the HAs at home. It can also be seen from the demographic indicators in Table S3 of the Supplementary Materials that this class had a majority of female respondents (58%), which is significantly higher in proportion when compared with other latent classes. Staehelin et al. [25] found gender as a determinant of non-regular HA use, where men were observed not to use the HAs regularly. Similarly, in this study, 51% of the females responding to questions related to taking off the HAs reported themselves as regular users compared to 27% of males. The regular users had higher hearing deficits indicated by higher mean better hearing ear PTA4 than participants in other classes. Laplante-Lévesque et al. [10] found a bivariate correlation between self-reported hearing disability and HA usage. This study also shows the effect of having a higher degree of hearing disability on HA usage patterns at a multivariate level. It was more likely that a user with an income between DKK 300,000 and 900,000 (EUR 40,000 and 120,000) would have a membership to Class 2 (higher probability of taking off the HAs in multiple situations). In addition, it is more likely to be in Class 3 (higher probability of taking off the HAs when at home) than in Class 1 (low probability of taking off the HAs) if the HA users have an income higher than DKK 900,000 (EUR 120,000).

Similarly, latent classes were also determined from the situations where HA users reported always putting on their HAs (implying that the default would be not to have it on). Here we can see that Class 2 was distinct from the other classes. Class 2 was more likely to put their HAs on for work. HA users with membership in Class 2 were more likely to be younger than the other classes and had (as would be expected) significantly better hearing (PTA4) as well as a higher proportion of interaural asymmetric HA users. Class 3 had a higher proportion of experienced HA users with higher hearing deficits (higher PTA4) than other classes. The members of Class 2 were predominantly still working and had a higher income (as would be expected) when compared to other classes, including regular users. Moreover, from the results, we can also see that Class 1 was predominantly a group of participants who are not part of the job market (retired), which may have influenced the HA usage pattern where the use of HAs was highly probable only in limited situations (such as watching TV, at home, and social interaction). Overall, the results of the LCA could suggest that the demand for HA was intended to resolve these specific situations or, with time, has proven only helpful in these situations. These behaviors could also evolve from the routine lifestyle of the HA users, as argued by Williger et al. [1].

The association of patterns of HA usage indicated by the class membership (situational non-use, situational use, and non-users) and self-reported HA outcomes was evident for all three outcomes (Factor 1, Factor 2, and total SSQ12). IOI-HA Factor 1 is linked to the introspection about HA use [16], where HA users score on the self-perceived benefit of using a HA. The results for both scenarios show (see Table 2 and Table 3) that participants who were situational non-users or users and non-users had a lower self-perceived benefit from using the HA than the regular users. Situational non-use, situational use, and non-use of HA also resulted in lower IOI-HA Factor 2 scores than the regular users. IOI-HA Factor 2 represents the perceived benefit of using HA for interaction with the outside world. The situational use and non-use of the HA also show a similarly significant effect on the total SSQ12 score, representing the functional benefit of using HA in specific situations linked to understanding speech in complex environments, spatial awareness, and quality of hearing. This could be in agreement with various studies [4,26,27,28] that found an association between higher usage of HA and satisfaction. However, various studies [9,27,29] also found that HA users with shorter HA usage time were also satisfied with HA use.

The results also suggested that participants with higher deficits may have lower benefits using HA in a complex listening environment, as indicated by lower IOI-HA Factor 2 and SSQ12 total scores. However, the positive coefficient for better hearing ear PTA4 for IOI-HA Factor 1 suggests that the higher the deficit, the higher the Factor 1 score. Similar results were found from the analysis of IOI-HA collected after two months of initial fit in the same project, as reported by Houmøller et al. [30]. This means that the significant effect of the hearing deficit on the IOI-HA Factor score follows the same trend as reported by the participants at the two-month follow-up. First-time HA users reported higher long-term outcomes with respect to IOI-HA Factor 1 and total SSQ12 scores compared to experienced HA users. The participants with PTA4-based symmetric HL also reported higher self-reported outcomes than participants with asymmetric HL. Ferguson et al. [31] found that self-efficacy toward HA rehabilitation was a predictor of satisfaction with hearing aids, in agreement with the results from this study where participants with higher self-efficacy (Motivation Q2) reported higher self-reported outcome scores. The presence of tinnitus also affected the self-reported outcome, with participants with tinnitus reporting lower outcomes in terms of IOI-HA Factor 2 and SSQ12.

The larger effect sizes concerning the class of HA usage patterns and adjusted coefficient of determination R2 were 0.36 and 0.38 for the IOI-HA Factor 1 models, suggesting reasonable clinical significance beyond the statistical significance. However, the effect sizes for IOI-HA Factor 2 and total SSQ12 in terms of HA usage were not as large as IOI-HA Factor 1, with an adjusted coefficient of determination R2 ranging from 0.12 to 0.17, implying a lower clinical significance despite the statistical significance.

### Limitations

Firstly, although our participants had a very heterogenous representation of HL and were recruited as part of standard clinical practice and not by advertisement, the usage patterns can be influenced by various factors such as culture, soundscapes, and many more. So, the generalizability of the results with respect to a population outside Denmark is unknown. However, the data-driven approach adopted in the study is universal and can be applied elsewhere. We also believe these responses could have resulted from their long-term routine or behavior, as they have used these HAs for over a year; in this case, even though the responses are likely based on most recent experiences, they could be representative of their experience in general, given that it has likely stabilized.

## 5. Conclusions

The study provides an understanding of different underlying patterns of HA usage amongst the adult Danish population. It established the relationship between the latent classes having distinct characteristics of HA use and the audiometric and socio-economic characteristics of the HA users. The results also emphasize the importance of regular use of HAs. Hearing care professionals can use these results during counseling to better understand HA’s situational usage and to emphasize the importance of regular HA usage to their patients/clients.

## Figures and Tables

**Table 1:** The labeled categories derived from the theme analysis of the free-hand text responses to the “Other” category in the questionnaires.

Categorized Situations	Description
Cultural Activity	It included descriptions about using or not using HA while in the cinema, theatre, concerts, listening to music, playing musical instruments, etc.
Outdoor Activity	The descriptions included hunting, sailing, gardening, golf club, etc.
Social Interaction	Interacting with a small group of people and attending meetings/lectures/talks.
Tinnitus	Situations when they experienced tinnitus.
Discomfort	Two aspects were described: (1) Physical discomfort caused due to sweating or itching and (2) Emotional discomfort due to fatigue or tiredness.
Fear of Losing	Descriptions included situations where there was a fear of losing the HA, for example, while playing with grandchildren and many more.
Noisy Situation	This included descriptions of machine noise, reverberation, and other natural noises.
Physical Activity	Description of situations included while at the gym, doing yoga, running, and other physical activities.
Sleep	During sleep.
Wet Condition	The description included situations related to being in the rain or at a swimming pool where there was a chance of HA getting wet.
When Alone	When alone at home or elsewhere.
Windy Condition	It included situations where a lot of wind noise was described.
With Many People	The description included situations such as being alongside many people or very large gatherings.
Non-use	Describing the situation that they had stopped using the HAs altogether.
Invalid Responses	Severely inconsistent responses.

**Table 2:** Stepwise multiple linear regression analysis of two IOI-HA Factor scores and total SSQ12 scores for situations where HA users always took off their hearing aids.

Predictors	IOI-HA Factor 1 Adj R^2^ = 0.36		IOI-HA Factor 2 Adj R^2^ = 0.12		Total SSQ12 Adj R^2^ = 0.15	
	Estimates [CI]	*p*-Value	Estimates [CI]	*p*-Value	Estimates [CI]	*p*-Value
(Intercept)	0.93 [0.40–1.46]	**0.001**	−0.14 [−0.73–0.46]	0.649	5.06 [4.63–5.50]	**<0.001**
Hearing aid use (Ref: Regular use)						
Class 1	−2.03 [−2.52–−1.53]	**<0.001**	−0.51 [−0.90–−0.11]	**0.012**	−0.17 [−0.46–0.11]	0.229
Class 2	−4.02 [−4.77–−3.26]	**<0.001**	−0.77 [−1.37–−0.17]	**0.012**	−0.44 [−0.88–−0.01]	**0.045**
Class 3	−3.13 [−3.90–−2.35]	**<0.001**	−0.53 [−1.15–0.09]	0.092	−0.47 [−0.92–−0.03]	**0.038**
Non-use	−8.88 [−9.95–−7.82]	**<0.001**	−2.74 [−3.58–−1.90]	**<0.001**	−0.55 [−1.16–0.05]	0.073
Age	−0.19 [−0.42–0.04]	0.102	NA		NA	
Gender, Male (Ref. Female)	NA		−0.26 [−0.61–0.10]	0.152	0.36 [0.10–0.62]	**0.006**
User Type, First time (Ref. Experienced)	NA		0.79 [0.38–1.20]	**<0.001**	0.54 [0.24–0.83]	**<0.001**
Better hearing ear PTA4	0.27 [0.04–0.51]	**0.024**	−0.30 [−0.51–−0.09]	**0.005**	−0.33 [−0.48–−0.18]	**<0.001**
Better hearing ear WRS	NA		0.23 [0.04–0.41]	**0.017**	0.23 [0.09–0.36]	**0.001**
PTA-based Symmetric HL (Ref: asymmetric HL)	0.58 [0.03–1.12]	**0.039**	0.62 [0.19–1.06]	**0.005**	0.60 [0.28–0.91]	**<0.001**
Tinnitus (Present) (Ref: not present)	NA		−0.51 [−0.95–−0.08]	**0.020**	−0.92 [−1.23–−0.61]	**<0.001**
Motivation Q1 score (Readiness)	NA		−0.27 [−0.47–−0.06]	**0.010**	NA	
Mean motivation Q2 score (Self-efficacy)	0.69 [0.47–0.91]	**<0.001**	0.24 [0.04–0.45]	**0.020**	0.21 [0.08–0.33]	**0.001**
Work status (Ref. Working) Not part of the job market	NA		0.11 [−0.29–0.51]	0.585	NA	
Not working			−0.96 [−1.89–−0.03]	**0.042**		
Income (in DKK) (Ref. 100,000–300,000) (EUR 12,500–40,000) under 100,000 (under EUR 12,500)	NA		NA		1.16 [−2.04–−0.28]	**0.010**
300,000–600,000 (EUR 40,000–80,000)					0.30 [−0.00–0.60]	0.052
600,000–900,000 (EUR 80,000–120,000)					0.32 [−0.10–0.73]	0.134
Above 900,000 (Above EUR 120,000)					0.81 [0.16–1.46]	**0.015**
Undisclosed					−0.20 [−0.57–0.18]	0.299

NA means that a specific variable was not part of the optimal model. Significance levels *p* < 0.05 are marked in bold. PTA4—Four-frequency air condition pure tone hearing threshold average; WRS—Word Recognition Score; HL—Hearing loss; PTA-based Symmetric HL—PTA4 difference of 10 dB or less than 10 dB between better and worst hearing ear; DKK—Danish kroner.

**Table 3:** Stepwise multiple linear regression analysis of two IOI-HA Factor scores and total SSQ12 scores for situations where HA users always put on their hearing aids.

Predictors	IOI-HA Factor 1 Adj R^2^ = 0.38		IOI-HA Factor 2 Adj R^2^ = 0.13		Total SSQ12 Adj R^2^ = 0.17	
	Estimates [CI]	*p*-Value	Estimates [CI]	*p*-Value	Estimates [CI]	*p*-Value
(Intercept)	0.33 [−0.24–0.89]	0.261	−0.06 [−0.66–0.53]	0.833	5.10 [4.68–5.53]	**<0.001**
Hearing aid use						
(Ref: Regular use)						
Class 1	−4.06 [−4.59–−3.54]	**<0.001**	−0.71 [−1.14–−0.28]	**0.001**	−0.31 [−0.62–−0.00]	0.047
Class 2	−2.24 [−3.13–−1.34]	**<0.001**	−0.67 [−1.41–0.06]	0.071	−0.44 [−0.97–0.09]	0.104
Class 3	−1.88 [−3.05–−0.71]	**0.002**	−1.40 [−2.34–−0.45]	0.004	−1.15 [−1.83–−0.47]	**0.001**
Non-use	−9.42 [−10.69–−8.15]	**<0.001**	−3.65 [−4.67–−2.63]	**<0.001**	−1.00 [−1.74–−0.26]	**0.008**
Age	−0.21 [−0.44–0.03]	0.080	NA		−0.11 [−0.26–0.03]	0.122
Gender, Male (Ref. Female)	NA		−0.32 [−0.67–0.03]	0.074	0.36 [0.11–0.62]	**0.005**
User Type, First time (Ref. Experienced)	0.46 [−0.05–0.97]	0.078	0.71 [0.30–1.12]	**0.001**	0.50 [0.20–0.79]	**0.001**
Better hearing ear PTA4	0.46 [0.21–0.71]	**<0.001**	−0.30 [−0.51–−0.09]	**0.005**	−0.31 [−0.46–−0.15]	**<0.001**
Better hearing ear WRS	NA		0.23 [0.05–0.42]	**0.014**	0.21 [0.07–0.34]	**0.003**
PTA-based Symmetric HL (Ref: asymmetric HL)	0.56 [0.03–1.09]	**0.040**	0.66 [0.23–1.09]	**0.003**	0.60 [0.29–0.91]	**<0.001**
Tinnitus (Present) (Ref: not present)	NA		−0.54 [−0.97–−0.11]	**0.013**	−0.99 [−1.30–−0.68]	**<0.001**
Motivation Q1 score (Readiness)	NA		−0.29 [−0.49–−0.08]	**0.006**	−0.15 [−0.29–0.00]	0.056
Mean motivation Q2 score (Self-efficacy)	0.56 [0.34–0.77]	**<0.001**	0.20 [−0.01–0.40]	0.062	0.25 [0.01–0.40]	**0.001**
Work status (Ref. Working) Not part of the job market	NA		0.04 [−0.37–0.46]	0.840	NA	
Not working			−0.97 [−1.91–−0.02]	**0.045**		
Income (in DKK) (Ref. 100,000–300,000) (EUR 12,500–40,000) under 100,000 (under EUR 12,500)	NA		NA		−1.19 [−2.07–−0.31]	**0.008**
300,000–600,000 (EUR 40,000–80,000)					0.28 [−0.02–0.58]	0.065
600,000–900,000 (EUR 80,000–120,000)					0.32 [−0.11–0.75]	0.145
Above 900,000 (Above EUR 120,000)					0.74 [0.09–1.40]	**0.026**
Undisclosed					−0.17 [−0.55–0.20]	0.361

NA means that a specific variable was not part of the optimal model. Significance levels *p* < 0.05 are marked in bold. PTA4—Four-frequency air condition pure tone hearing threshold average; WRS—Word Recognition Score; HL—Hearing loss; PTA-based Symmetric HL—PTA4 difference of 10 dB or less than 10 dB between better and worst hearing ear; DKK—Danish kroner.

## Data Availability

The data are not publicly available due to patient data privacy.

## Supplementary Materials

The following supporting information can be downloaded at: https://www.mdpi.com/article/10.3390/audiolres13020021/s1, Figure S1: Flow chart showing participant inclusion and exclusion criteria for further analysis of responses to the question regarding the situations where participants always take off their hearing aids (HAs); Figure S2: Flow chart showing participant inclusion and exclusion criteria for further analysis of responses to the question regarding the situations where participants always put on their hearing aids (HAs); Table S1: Goodness of fit table for Situations where hearing aid (HA) users preferred to take off their HAs; Table S2: Goodness of fit table for Situations where hearing aid (HA) users preferred to put on their HAs; Table S3: The covariate distribution across latent classes of situations where users always preferred to take off their hearing aids (HAs); Table S4: The covariate distribution across latent classes of situations where users always preferred to put on their hearing aids (HAs).
