# Supplementary material for: Self-Reported Hearing-Aid Use Patterns in an Adult Danish Population
Source: Audiol Res. 2023 Mar 27;13(2):221–35. doi: 10.3390/audiolres13020021 (PMC10135679; doi:10.3390/audiolres13020021)
Supplement: Supplementary file 1 [file audiolres-13-00021-s001.zip › audiolres-2222238-supplementary.pdf]

Figure S1: Flow chart showing participant inclusion and exclusion criteria for further analysis of responses to the question regarding the situations where participants always take off their hearing aids (HAs).

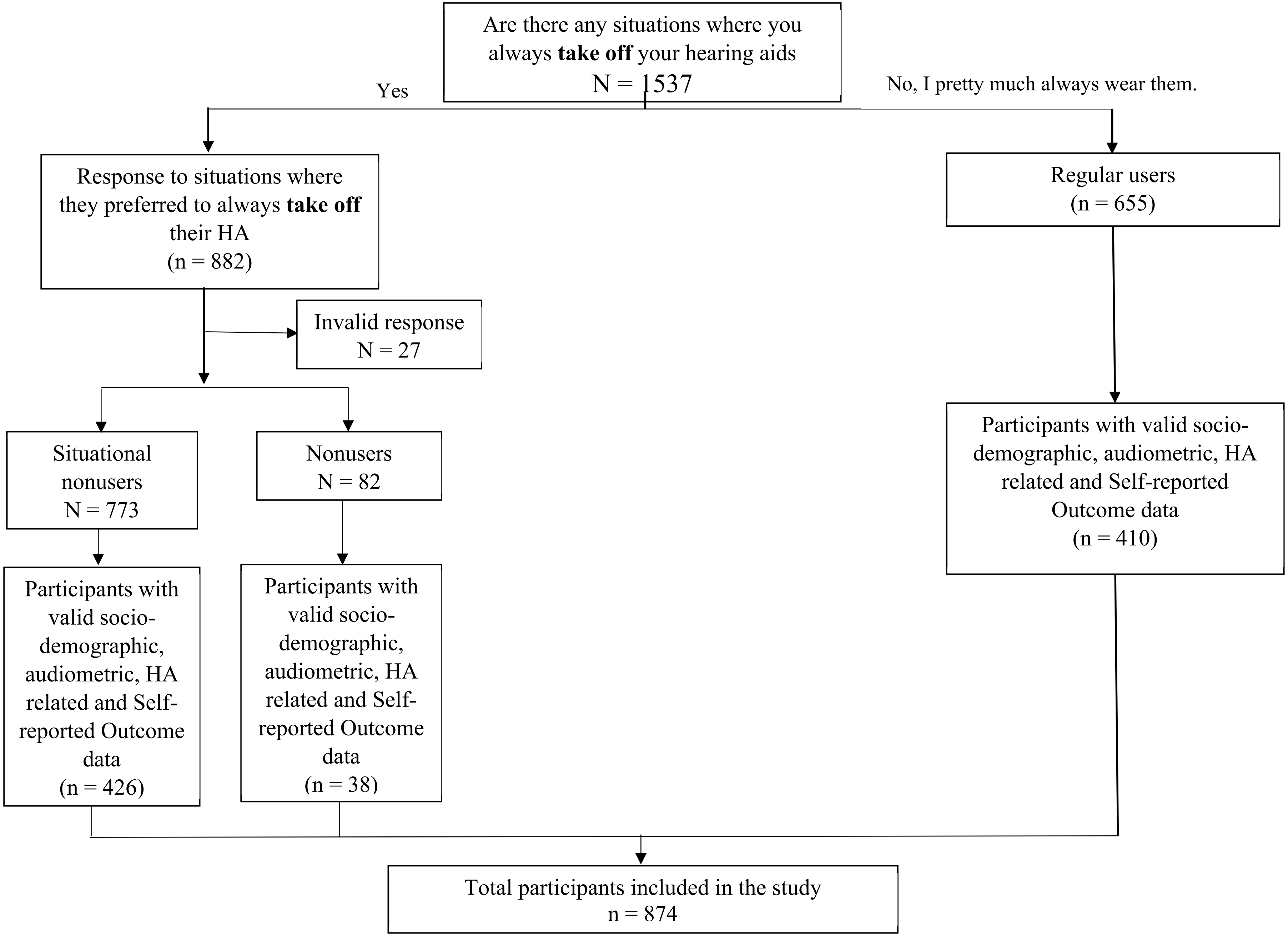

Figure S2: Flow chart showing participant inclusion and exclusion criteria for further analysis of responses to the question regarding the situations where participants always put on their hearing aids (HAs).

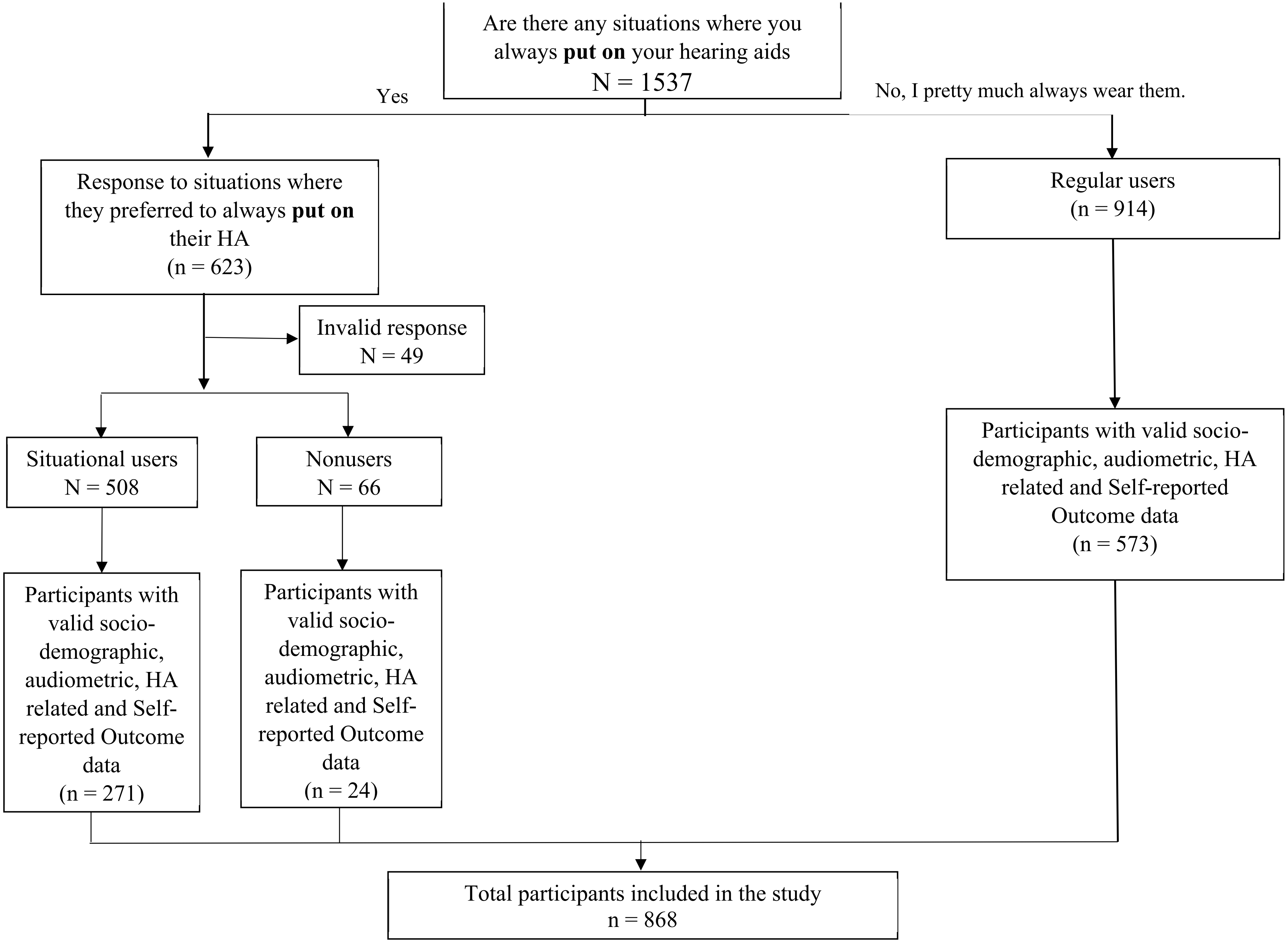

Table S1: Goodness of fit table for Situations where hearing aid (HA) users preferred to take off their HA:

	logLik	AIC	CAIC	BIC	Entropy	Res.Df	G^2	p
2 Classes	-2150.17	4382.34	4589.57	4548.57	0.85	384	856.54	0.00
3 Classes	-2101.40	4326.81	4640.18	4578.18	0.83	363	759.00	0.00
4 Classes	-2066.37	4298.74	4718.26	4635.26	0.86	342	688.93	0.02
5 Classes	-2036.39	4280.79	4806.45	4702.45	0.89	321	628.98	0.00
6 Classes	-2006.20	4262.40	4894.20	4769.20	0.87	300	568.59	0.00

Table S2: Goodness of fit table for Situations where hearing aid (HA) users preferred to put on their HAs

	logLik	AIC	CAIC	BIC	Entropy	Res.Df	G^2	p
2 Classes	-1153.48	2360.95	2485.21	2458.21	0.92	243	453.58	0.00
3 Classes	-1127.90	2337.79	2526.48	2485.48	0.95	229	402.42	0.00
4 Classes	-1107.34	2324.68	2577.80	2522.80	0.86	215	361.31	0.00
5 Classes	-1084.49	2306.97	2624.52	2555.52	0.94	201	315.61	0.00
6 Classes	-1075.46	2316.92	2698.90	2615.90	0.90	187	297.56	0.00

Appendix C

Table S3: The covariate distribution across latent classes of situations where users always preferred to take off their hearing aids (HAs). The p-values were computed from χ2-analyses for categorical variables or Kruskal–Wallis test for continuous variables. Fisher's exact test was used for categorical variables when the expected frequency was less than 5. Significance levels $p<0.05$ are marked in bold.

Covariates	Regular users N = 410	Situational Non-users			Non-users N=38	*p*
		Class 1 N=264	Class 2 N=85	Class 3 N=77		
Mean age, years (±SD)	66 (10)	66 (11)	66 (10)	65 (11)	64 (11)	0.41
Gender (Female), n	168	77	24	45	17	**<0.001*** **
User Type (First-time users), n	264	184	76	57	31	**<0.001*** **
Mean better hearing ear PTA4, dB HL(±SD)	39 (12)	36 (13)	35 (10)	37 (11)	32 (10)	**<0.001*** **
Mean better hearing ear WRS, % (±SD)	89 (15)	90 (15)	92 (9)	92 (11)	92 (8)	0.66
PTA-based asymmetry, n	81	52	26	4	6	**0.002** **
Tinnitus (Present), n	69	53	23	15	9	0.24
Mean motivation Q1 score (Readiness) (±SD)	8.5 (1.7)	8.1 (1.9)	7.7 (2.1)	8 (2)	7.7 (1.7)	**<0.001*** **
Mean motivation Q2 score (Self-efficacy) (±SD)	8.6 (1.6)	8 (2.1)	7.5 (2)	8 (2)	7.5 (2.2)	**<0.001*** **
Work status, n						0.50
Not part of the job market	292	192	62	51	23	
Not working	14	8	3	6	1	
Working	104	64	20	20	14	
Household members, n						0.15
Alone	72	46	21	24	10	
Two members	303	197	58	48	27	
Three members and above	35	21	6	5	1	
Income (in DKK), n						**0.001** **
Under 100,000	9	5	1	2	0	
100,000 to 300,000	113	88	15	25	14	
300,000 to 600,000	152	89	45	22	12	
600,000 to 900,000	44	38	5	11	9	
900,000 and above	13	7	6	8	0	
Don't know / Undisclosed	79	37	13	9	3	

Table S4: The covariate distribution across latent classes of situations where users always preferred to put on their HAs. The p-values were computed from χ2 -analyses for categorical variables or Kruskal–Wallis test for continuous variables. Fisher's exact test was used for categorical variables when the expected frequency was less than 5. Significance levels p<0.05 are marked in bold.

Covariates	Regular users N= 573	Situational Users			Non-users N=24	
		Class 1 N=189	Class 2 N=54	Class 3 N=28		*p*
Mean age, years (±SD)	67 (10)	68 (9)	55 (13)	64 (13)	62 (13)	**<0.001*****
Gender (Female), n	219	62	23	14	13	0.13
User Type (First-time users), n	370	160	41	15	20	**<0.001*****
Mean better hearing ear PTA4, dB HL(±SD)	38 (13)	35 (11)	32 (11)	40 (12)	33 (10)	**<0.001*****
Mean better hearing ear WRS, % (±SD)	90 (15)	91 (11)	94 (11)	90 (11)	94 (7)	0.12
PTA-based asymmetry, n	116	34	7	6	6	0.65
Tinnitus (Present), n	102	44	13	6	5	0.47
Mean motivation Q1 score (Readiness) (±SD)	8.5 (1.7)	7.6 (2)	7.9 (1.8)	8.4 (2.1)	8 (2.3)	**<0.001*****
Mean motivation Q2 score (Self-efficacy) (±SD)	8.6 (1.6)	7.4 (2.2)	7.9 (2)	8 (1.9)	7.2 (2.5)	**<0.001*****
Work status, n						**<0.001*****
Not part of the job market	421	154	8	17	14	
Not working	16	6	6	1	1	
Working	136	29	40	10	9	
Household members, n						**0.006****
Alone	107	141	11	8	4	
Two members	418	7	32	20	17	
Three members and above	48	41	11	0	3	
Income (in DKK), n						**<0.001*****
Under 100,000	10	5	2	0	0	
100,000 to 300,000	170	58	7	9	8	
300,000 to 600,000	212	74	17	8	4	
600,000 to 900,000	55	23	19	3	7	
900,000 and above	20	4	7	2	1	
Don't know / Undisclosed	10	25	2	6	4	
